# The Impact of Radiotherapy on Frailty in Patients Aged 65 and Over

**DOI:** 10.7759/cureus.46351

**Published:** 2023-10-02

**Authors:** Zeliha Güzelöz, Umut Gök Balcı

**Affiliations:** 1 Radiation Oncology Department, University of Health Sciences, Tepecik Training and Research Hospital, İzmir, TUR; 2 Palliative Care Clinic, University of Health Sciences, Tepecik Training and Research Hospital, İzmir, TUR

**Keywords:** edmonton frail scale, frail elderly, frailty, aged, radiotherapy

## Abstract

Introduction and objective

Frailty is characterized by the body's increased susceptibility to stressors due to aging and a concurrent decline in its resilience. A dominant hypothesis suggests that oncological interventions may amplify this vulnerability. Consequently, elderly individuals with cancer may pose challenges for conventional treatments. This research sought to assess the effects of radiotherapy (RT) on the frailty of elderly cancer patients by utilizing the Edmonton Frail Scale (EFS).

Methods

This research was designed as a prospective observational study. Patients aged 65 and older, receiving treatment at the radiation oncology clinic, were asked to complete the EFS form both before and at the end of their RT course. The scores achieved before and after the RT were obtained, and the medical histories of the patients were recorded. The difference between the pre- and post-RT scores was calculated by subtracting the post-RT score from the pre-RT score to determine any increase or decrease in the score. For the statistical evaluations, a suite of tests (including the Shapiro-Wilk test, Kolmogorov-Smirnov test, paired sample t-test, independent t-tests, analysis of variance {ANOVA}, and Pearson correlation) were implemented. All analyses were conducted using the Statistical Package for Social Sciences (SPSS) software (version 29.0) (IBM SPSS Statistics, Armonk, NY).

Results

From April 2021 to August 2023, a total of 121 patients participated in the study. Out of these, 81 underwent assessments both pre- and post-RT. The median age was 73; the median follow-up period was five months. The predominant diagnoses included prostate cancer, breast cancer, and gynecological malignancies. A significant proportion of patients was diagnosed with stage IV cancer and underwent palliative RT. Post-RT evaluations revealed a decline in scores for some patients, while an increase was observed for others. Certain score variations were statistically significant. Moreover, an inverse correlation was discerned between the RT dose and fraction number and the post-RT EFS score.

Conclusion

Our research confirmed that a substantial number of patients either experienced a decrease or maintained stability in their EFS scores after RT. This observation suggests that RT might not exacerbate frailty in the elderly in the short term. Nevertheless, to elucidate the long-term impact of RT on frailty, there is a pressing need for a comprehensive assessment correlating EFS scores with survival rates.

## Introduction

Frailty can be defined as the increased vulnerability of the aging body to stressor factors. This state is associated with aging in multiple organ systems [1,2]. Among geriatric syndromes that cause increased mortality and morbidity in elderly patients, frailty is one of the most essential concepts to address. The energy loss, loss of strength, and weight loss that begins with frailty also affect the patient's psychological functions, leading to the person transitioning into a weak profile [3].

Comprehensive geriatric assessment (CGA) is an evaluation method considered the gold standard for holistically assessing elderly patients in terms of their physical, mental, metabolic, nutritional, and social states [4]. Through CGA, we determine whether patients can tolerate the intended treatments and whether they need supportive therapies. The existing comorbidities in this age group can worsen the course of cancer during radiotherapy (RT) and may influence prognosis and treatment tolerance.

Frailty is a parameter within the CGA [5,6]. It can be assessed using various tests. The G-8 test is one of the most frequently used frailty tests in daily practice [7-9]. The Edmonton Frailty Scale (EFS) is another widely used, brief test with geriatric validation. This test assesses the patient in terms of frailty [10]. The test, consisting of 11 questions, provides information about cognitive status, general health, functional independence, social support, medication use, nutritional status, mood, urinary continence, and mobility [11]. Based on the scoring, patients are categorized as fit, vulnerable, mildly frail, moderately frail, or severely frail. An assessment using the EFS can be completed in less than five minutes, providing insights, especially regarding the postoperative state and hospitalization duration after surgical procedures [12]. From this perspective, the EFS initially used for surgical evaluations has been adopted in various disciplines over time [13-15]. In Røyse et al.'s study, it was mentioned that EFS could predict mortality after RT [13].

Approximately 70% of oncology patients undergo RT at some stage of their treatments. In our daily practice, when evaluating an elderly patient whom we believe is frail, we may opt for less aggressive treatment out of concern that the patient might not tolerate it well [16]. This can prevent patients from receiving the treatment they deserve. However, the International Society of Geriatric Oncology (SIOG) recommends that elderly patients should be detailed and realistically evaluated to ensure the provision of standard treatments as much as possible [7]. Stemming from this idea, in our study, we aimed to use the EFS to answer the following question: does RT increase frailty in elderly geriatric patients?

## Materials and methods

Study design and patients

The study was a prospective and observational study. The study protocol was approved by the ethics committee of the University of Health Sciences, Tepecik Training and Research Hospital (approval number: 2021/02-65). Patients referred to the radiation oncology clinic were asked to participate in the study on a voluntary basis. Consent forms were obtained from all patients who agreed to participate. All patients aged 65 years and over who were indicated for RT and had a Karnofsky Performance Score (KPS) of ≥50 were included in the study. Patients with a KPS below 50 assessed by the study's radiation oncologist and those whose mental and cognitive functions were impaired if they could not answer questions were excluded from the study. Patients completed two EFS forms, one before and one after RT. During the first visit of patients applying to the outpatient clinic for RT, they were informed about the study. A consent form and a pre-radiotherapy EFS form were given. The EFS form was completed either by the patient or with the help of a relative. A form containing medical information about the patients, such as age, weight, height, diagnosis, stage, RT field, RT dose, and chemotherapy (CT) details, was completed by the doctor during the patient's medical history and while planning RT. As the study began during the pandemic, patients were also asked if they had contracted COVID-19. Patients who were uncertain about having COVID-19 due to not being tested marked the "unknown" option on the form. RT was initiated no later than a week after this procedure. During RT, patients were called in for weekly examinations. Routine full blood counts and biochemical evaluations were performed. Patients were asked to complete the second EFS form either on the last day of RT or at most within the first week following RT. The total score from the 11 questions provided on the form before and after RT was calculated. In the study, the total score from the pre-RT EFS form was termed Pre-RTEFS, whereas the post-RT EFS form was termed Post-RTEFS.

Primary assessments and Edmonton scale

Patients' cancer diagnoses were made according to the International Classification of Diseases, Tenth Revision (ICD-10). Staging was conducted on the basis of the American Joint Committee on Cancer tumor, lymph node, and distant metastasis (TNM) staging system (eighth edition, 2017). Patient performance was assessed with the KPS. Patients' medical information was obtained during medical history-taking.

Frailty assessment in patients was conducted using the EFS, which is a test consisting of 11 questions spread across nine main sections. It assesses cognitive functions, general health status, social support, functional independence, medication usage, nutritional status, mood, urinary incontinence, and mobility. Scores range from 0 to 2 for the answers. A maximum of 17 points can be attained in total. As the score increases, frailty also intensifies. Based on the score, patients were classified as fit (0-3), vulnerable (4-5), mild frailty (6-7), moderate frailty (8-9), and severe frailty (≥10) [11].

Radiotherapy

RT was planned for all patients by performing a tomography administered by using either the volumetric modulated arc therapy (VMAT) or intensity-modulated radiotherapy (IMRT) techniques. All patients received treatment using the same Varian VitalBeam© device (Palo Alto, CA). Every treatment day was monitored using a port film or cone beam tomography.

Statistics

The final follow-up status of the patients was identified from hospital and national data systems. The follow-up duration was calculated by subtracting the start date of RT from the last follow-up date. The difference between the Pre-RTEFS and Post-RTEFS scores was calculated by subtracting the Post-RTEFS score from the Pre-RTEFS score to determine any increase or decrease in the score. This change in value, expressed as "ScD" or score difference, indicates that a negative value suggests an increase in the score after RT, whereas a positive value suggests a decrease in the score. The Shapiro-Wilk and Kolmogorov-Smirnov tests were employed to examine the normal distribution of data, depending on their appropriateness. Descriptive statistics related to categorical variables are presented using counts and percentages. For data that followed a normal distribution, the mean and standard deviation were provided, whereas for data that did not, the median and interquartile range (IQR) were detailed. The scale scores taken before (Pre-RTEFS) and after (Post-RTEFS) the treatment were analyzed using a paired sample t-test. Continuous variables were subjected to independent sample t-tests and analysis of variance (ANOVA), based on the specific characteristics of the data. Pearson correlation analyses were also performed to explore the relationships between continuous variables. The data were evaluated using the Statistical Package for Social Sciences (SPSS) (version 29.0) software (IBM SPSS Statistics, Armonk, NY). A p-value of less than 0.05 was considered significant.

## Results

Between February 2021 and August 2023, the EFS was administered before and after RT to 121 patients who met the study criteria. From these, 81 patients who correctly and completely filled out both scales were considered, resulting in the analysis of 162 EFS tests.

Twenty percent of the patients (n=16) were aged 80 or above. The median age was determined to be 73 (ranging from 65 to 97), and the median body mass index (BMI) was 25.2 (ranging from 14.7 to 43.9). The average follow-up duration was five months (ranging from one to 30 months). The descriptive characteristics of the participants are provided in Table 1.

**Table 1:** Demographic characteristics of the patients

Characteristics	n (%)
Age	
65-74	51 (63%)
75-84	24 (30%)
≥85	6 (7%)
Gender	
Female	35 (43%)
Male	46 (57%)
COVID-19-positive	
Yes	8 (10%)
No	57 (70%)
Unknown	16 (20%)
Comorbid illness	
Yes	84 (80%)
No	16 (20%)
Status at follow-up	
Alive	58 (72%)
Deceased	16 (20%)
Unreachable	7 (8%)
Diagnosis	
Prostate cancer	24 (30%)
Breast cancer	11 (13%)
Gynecological cancers	8 (10%)
Lung cancer	7 (9%)
Rectum cancer	6 (7%)
Lymphoma	5 (6%)
Esophagus cancer	4 (5%)
Skin tumor	4 (5%)
Others	12 (15%)
Stage	
Stage I	12 (15%)
Stage II	12 (15%)
Stage III	21 (26%)
Stage IV	33 (41%)
Relapse	3 (3%)

Patients are classified into five groups based on scores they received on the EFS to assess their frailty levels: 0-3 points are defined as fit, 4-5 points as vulnerable, 6-7 points as mild frail, 8-9 point as moderate frail, and ≥10 as severe frail. The distribution of patients according to the EFS classification before and after RT is shown in Table 2.

**Table 2:** Distribution of patients based on the EFS EFS, Edmonton Frail Scale; RT, radiotherapy

EFS classification	Before RT, n (%)	After RT, n (%)
Fit (0-3 points)	24 (30%)	21 (26%)
Vulnerable (4-5 points)	23 (28%)	26 (32%)
Mild frail (6-7 points)	16 (20%)	14 (18%)
Moderate frail (8-9 points)	12 (15%)	10 (12%)
Severe frail (≥10 points)	6 (7%)	10 (12%)

RT was administered at the doses recommended by the guidelines based on the indications. Patients who had palliative RT received doses of 10×3 Gy, 12×2 Gy, 15×2.5-3 Gy, 5×4 Gy, and 1×8 Gy. The details of the RT administered to the patients are shown in Table 3.

**Table 3:** Details of RT RT: radiotherapy

Purpose of RT	n (%)
Palliative	30 (37%)
Neoadjuvant	12 (15%)
Adjuvant	21 (26%)
Definitive	16 (20%)
Salvage	2 (2%)
Median RT dose	45 Gy (range: 4-78)
Median number of RT fractions	25 days (range: 1-39)

Of the patients, 60.5% (n=49) were not administered CT, 18.5% (n=15) were administered adjuvant CT, 13.6% (n=11) received concurrent CT, and 5% (n=4) were administered neoadjuvant CT.

Post radiotherapy, scores decreased in 31 patients, increased in 21 patients, and remained the same in 29 patients. The Pre-RTEFS mean score was 5.75±2.6, and the Post-RTEFS mean score was 5.51±2.8 (p=0.3). For the fifth question evaluating social support status, the pre-treatment mean score was 0.3±0.5, and the posttreatment mean score was 0.18±0.45, which showed statistical significance (95% confidence interval {CI}: 0.16-0.23, p=0.024).

Correlations between Pre-RTEFS and Post-RTEFS with age, BMI, RT dose, number of RT fractions, and follow-up duration were examined. The Post-RTEFS score was found to be negatively correlated with the RT dose and the number of RT fractions (r=-0.33, p=0.03; r=-0.31, p=0.04, respectively). As the RT dose and fraction number increased, the Post-RTEFS score decreased. No significant correlation was observed with other variables.

There was no significant relationship between ScD and age, gender, having experienced COVID-19, BMI, the presence of comorbid diseases, RT dose, follow-up duration, and survival status. Evaluating ScD with CT information, neoadjuvant CT patients (mean, -2.0±2.7; 95% CI: -6.3 to -2.3) showed significantly lower scale scores compared with both adjuvant CT and concurrent CT patients (p=0.029 and 0.043, respectively).

In subgroup analysis, the 20% segment (n=16) of our study, which included six patients classified as "very elderly" by the World Health Organization, showed no significant difference in terms of ScD when compared with patients under 80 years of age.

## Discussion

Currently, 60% of all cancers are observed in individuals over the age of 65. In making oncological treatment decisions and evaluating side effects, the functional status of the patient is more important than their chronological age. CGA helps clarify functional and physical conditions and psychological and social status when deciding on palliative RT. A study conducted in 2017 indicated that a systematic evaluation of geriatric areas is essential to assist oncologists in identifying frail patients with low survival rates [17]. In the study by Røyse et al., the EFS score was found to predict overall survival for patients who underwent RT (p<0.001; hazard ratio (HR), 1.20; 95% confidence interval {CI}, 1.10-1.30) [13].

In our study, 20% of the patients were 80 years or older, with a median age of 73. In the study conducted by Keenan et al., involving cancer patients receiving RT, 63 volunteer patients were included, and the median age was determined to be 74 [18]. Another study involving 301 patients reported a median age of 73.6, and it also focused on cancer patients receiving RT [13]. All three studies that used the EFS were similar in terms of age groups.

Looking at the types of cancer in our patients, prostate cancer was the most common at 30%. Given that our study group was elderly and 57% were male, this result was expected.

Major comorbidities can worsen the course of cancer during RT, affecting prognosis and treatment tolerance; cardiovascular diseases, diabetes, kidney failure, cognitive impairment, depression, anemia, chronic infections, osteoporosis, and pressure ulcers are the most commonly observed diseases [19]. Hypertension was the most common comorbidity in our patients at 32.1%, followed by diabetes mellitus, coronary artery disease, and multiple comorbidities; however, no statistical effect on frailty was detected.

Because our study coincided with the COVID-19 pandemic, patients were asked whether they had contracted COVID-19 or not. In the elderly age, the COVID-19 pandemic has more severe effects and increases the mortality rate [20]. Hence, the impact of the pandemic was considered a factor that could affect all geriatric assessment criteria for these patients, and 70% reported that they did not contract the disease. No statistical significance was found in the frailty assessments of those who contracted the virus and those who were unsure about it.

In the current study, 60.5% of the patients did not receive concurrent chemotherapy, whereas 18.5% received adjuvant chemotherapy, 13.6% received concurrent chemotherapy, and 5% received neoadjuvant chemotherapy. A study published in 2015 stated that more than half of elderly cancer patients have pre-frailty or frailty, and these patients are at a high risk of chemotherapy intolerance, postoperative complications, and mortality [5]. When evaluating ScD with chemotherapy situation, the scale scores of patients receiving neoadjuvant chemotherapy were significantly lower than those receiving both adjuvant and concurrent chemotherapy (mean, 2.0±2.7; 95% CI, -6.3 to -2.3) (mean post-RT score>mean pre-RT score) (p=0.290 and 0.043, respectively). Based on these results, we interpreted it as neoadjuvant chemotherapy affecting the Edmonton score negatively and increasing frailty.

After RT, a decline in scores was observed in 31 patients and an increase in 21 patients, while the scores of 29 patients remained unchanged. It was noted that as the RT dose and the number of fractions increased, the Post-RTEFS scores decreased. It was determined that patients treated with higher doses exhibited a decrease in their post-RT scores, indicating a reduction in frailty. We believe that this may be attributed to effective symptom palliation through treatment. Obviously, the good performance of the patient group eligible for radical RT might be another reason for this observation. RT is an effective treatment method with relatively manageable tolerability. Because of easier patient adaptation and reduced toxicity compared with CT, RT and other supportive palliative therapies can be utilized as the single treatment modality. During the development of a personalized RT plan, considering the treatment-associated risks and benefits, performance status, and treatment options is vital, especially given the high probability of altering the biological behaviors of tumors in the elderly population [21]. The primary goal in elderly patients should be to complete RT in the shortest time with minimal toxicity [3]. In suitable patients, even if there is an increased risk of local and systemic toxicity, RT can be administered alongside CT [22]. To enhance tolerance and prevent a treatment interruption that could adversely affect outcomes, the CT dosage may also be reduced. However, for patients with severe functional limitations, alternative treatment approaches such as standalone RT or supportive therapies should be considered [19]. In addition, during the treatment process, the expectations of the patient and their caregivers regarding the treatment must be considered [21].

In the fifth question evaluating the status of social support, statistical significance was identified. This indicates a decrease in the perceived social support by the patient after treatment. In the study, based on elderly individuals' communication frequency with their children, it was determined that the difference in vulnerability level stemmed from the group that seldom communicated with their children. In this group, vulnerability levels were higher than those in others [23]. Comparing our findings with the study conducted by Son et al., it was found that elderly individuals who often or seldom communicated with close friends and family members had a higher vulnerability level than those who communicated moderately. Social support encompasses the material and emotional support provided by spouses, families, and friends. In our study, it is presumed that while there should be an anticipated increase in social attention as the treatment began, this expectation was not met [23].

In the subgroup analysis of our study, which constituted 20% (n=16) including six patients classified as "very elderly" by the World Health Organization, no significant difference was observed in terms of ScD between patients aged 80 and above and those below 80. Despite some individuals being vulnerable in their 60s or 70s, others remain vibrant, healthy, and active beyond 80. Schuurmans et al. demonstrated that frailty is a better indicator of a decrease in self-management ability than chronological age [24]. Our study has also yielded results that support this.

This study is not without its limitations. Notably, while RT is often the primary treatment choice for elderly cancer patients not suitable for surgery or chemotherapy, the guidelines for this demographic remain inadequate. Elderly patients have been frequently excluded from many randomized and prospective studies, leading to a limited body of literature on this topic [3,7,16]. Consequently, the scope of our research was narrowed due to the lack of standardized treatments. Furthermore, the preferences of elderly patients and their caregivers toward shorter-duration treatments have necessitated that this study be conducted within a more specific population. The data collection process was protracted because of the impact of the pandemic.

Despite the aforementioned limitations, this study offers valuable insights in an area of the literature that infrequently addresses RT and frailty. Existing literature indicates that the EFS test is typically conducted before treatment; however, in our study, evaluations were conducted both pre- and post-RT.

## Conclusions

Contrary to assumptions, RT may not worsen frailty in the elderly. Even at radical doses, RT has the potential to decrease frailty in suitable patients. As such, RT can be safely administered to elderly patients. These findings warrant further exploration of the long-term effects of RT on frailty and its relationship with survival rates, offering critical insights for the treatment of elderly cancer patients.
